# Cloacal type defect of the anal canal following an obstetric anal sphincter trauma

**DOI:** 10.1002/ccr3.4309

**Published:** 2021-05-24

**Authors:** Sofoklis Stavros, Ioannis K. Papapanagiotou, Dimitrios Zacharakis, Kyriaki Migklis, Rafail Mantzioros, Ekaterini Domali, Ioannis Chatzipapas, Peter Drakakis, Alexandros Rodolakis

**Affiliations:** ^1^ 1st Department of Obstetrics and Gynecology, University of Athens General Hospital “Alexandra” Athens Attiki 11528 Greece

## Abstract

Neglected severe obstetric anal sphincter injuries may result in fecal incontinence. It is of paramount importance to identify such injuries at the time of vaginal delivery and have appropriate surgical training for optimal anatomical restoration of the perineal structures.

## Graphical abstract

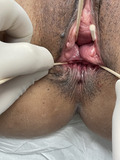

## 1 CLINICAL IMAGE

Cloacal type defect of the anal canal following an obstetric anal.

### 1.1 Question

What is the cause of this condition and how can be prevented?

### 1.2 Answer

**FIGURE 1:**
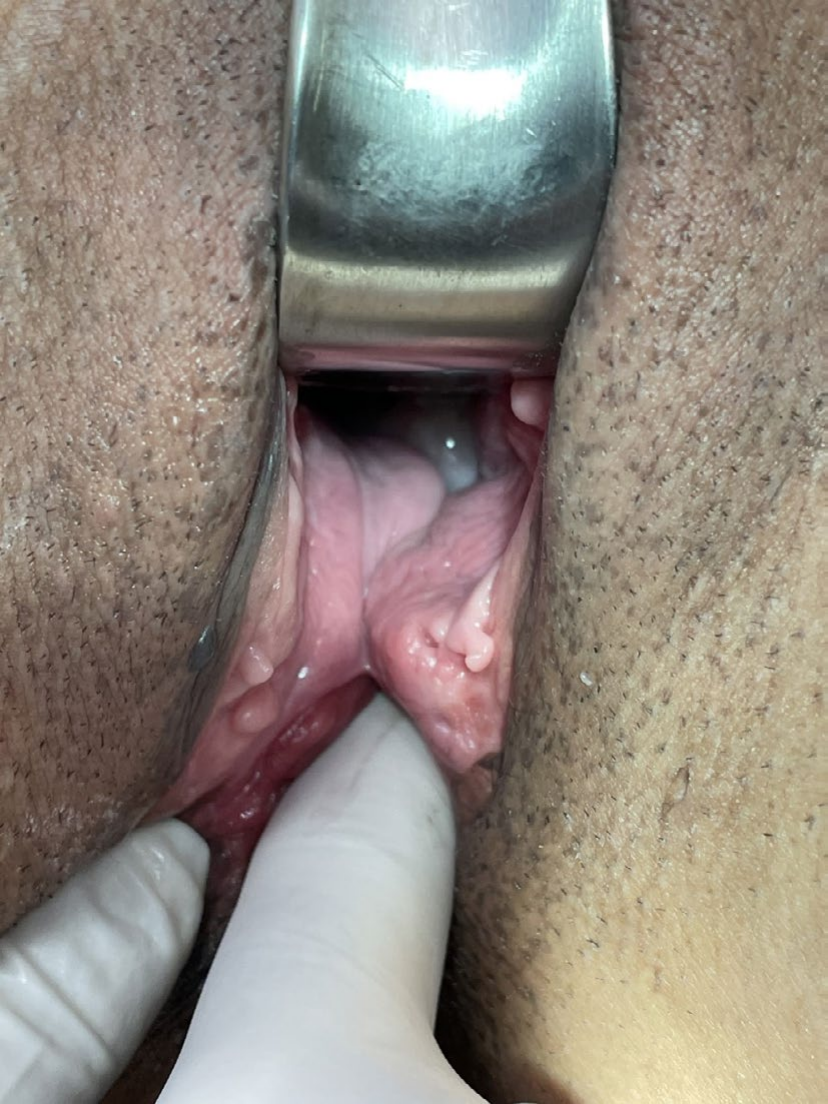
Digital rectal examination exposed a very large sphincteric defect, while both the resting tone and the squeeze contraction were completely absent

A 39‐year‐old woman (para 4 and gravida 4) presented to the Gynecological Outpatient Clinic with symptoms of superficial dyspareunia and anal incontinence. The onset of these symptoms occurred immediately after her last home‐vaginal delivery three years ago, in her country of origin. Clinical examination revealed absence of the perineal body and of the corrugator cutis. Digital rectal examination exposed a cloacal type defect of the anal canal in the distal posterior vaginal wall. Both resting tone and squeeze contraction of the anal sphincter were completely absent. The patient was referred to a colorectal specialist for further management (Figure 1).

**FIGURE 2:**
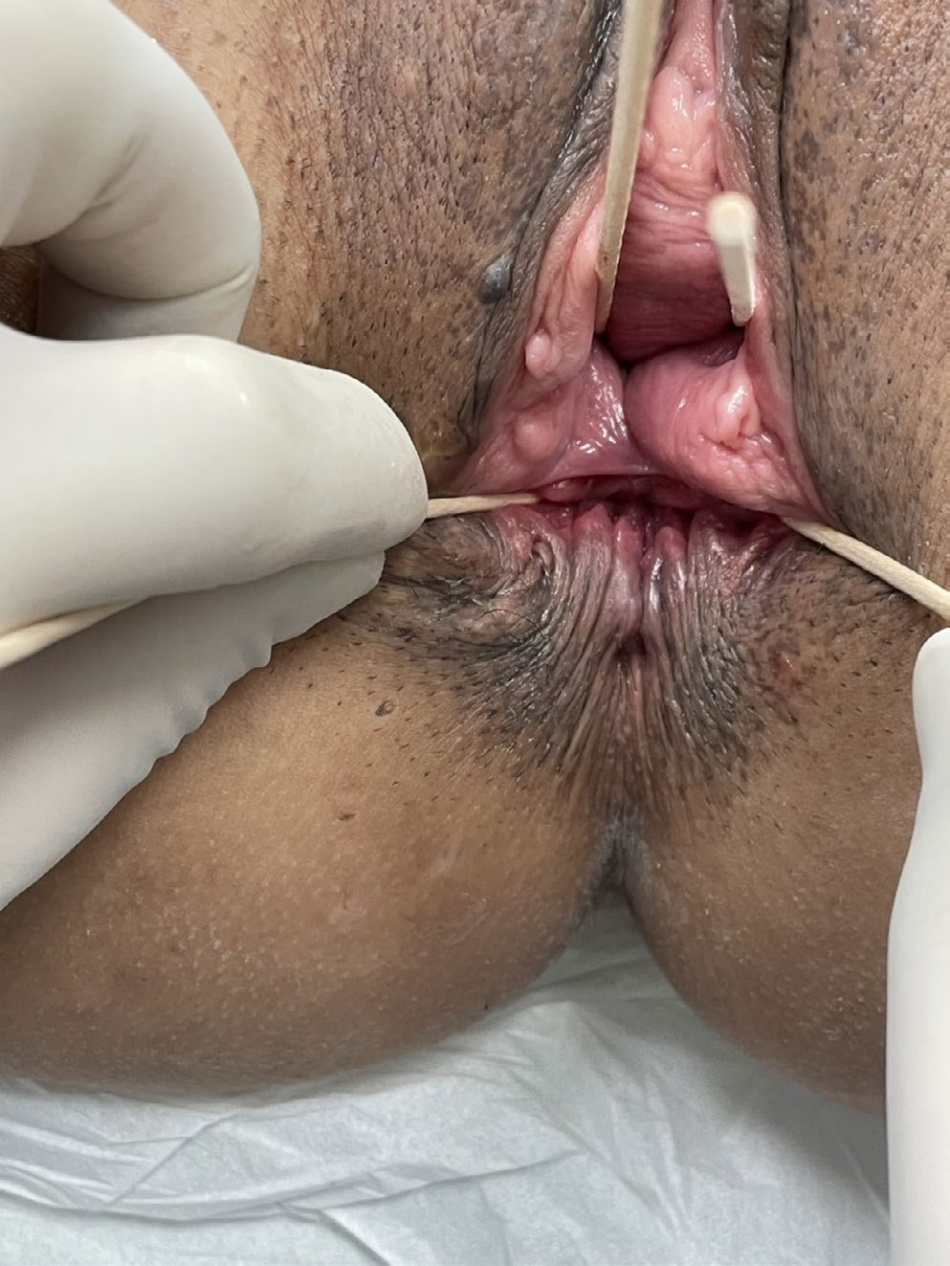
Anal inspection revealed absence of the perineal body, the corrugator cutis ani and an off‐site aperture of the anal canal in the posterior proximal vaginal surface

Obstetric anal sphincter injury (OASIS) is a relatively common complication of vaginal deliveries. The incidence of OASIS varies in the literature widely, reflecting wide variations in obstetric practice and inaccurate reporting related to training of doctors and midwives.^1^ Risk factors include maternal (primiparity, age, maternal diabetes, and infibulation), delivery (operative vaginal delivery, episiotomy, and shoulder dystocia), and infant (birthweight >4 kgr, malpresentation, and postmaturity) characteristics (Figure 2).

Severe OASIS may result in various complications such as anal incontinence, severely affecting physical and emotional well‐being of women. It is of paramount importance to have appropriate training to identify and manage such injuries at the time of vaginal delivery. ^2^

## CONFLICT OF INTEREST

None declared.

## AUTHOR CONTRIBUTIONS

SS: made substantial contribution to acquisition of data. IKP: made substantial contribution to conception, analyzing, and drafting the manuscript. DZ: contributed in analyzing data and revising the manuscript. KM: contributed in acquisition of the data. RM: contributed in acquisition of the data. ED: contributed in analyzing data. IC: revised the manuscript. PD: agreed to be accountable for all aspects of the work. AR: gave final approval of the version to be published.

## ETHICAL APPROVAL

Patient consent has been collected. The Ethics Committee of the Hospital has approved this Clinical Image.

## Data Availability

Data sharing not applicable‐no new data generated‐the article describes entirely an obstetrical complication.
